# Psychiatric morbidity among ICU patients at a general hospital in India

**DOI:** 10.6026/973206300211438

**Published:** 2025-06-30

**Authors:** S. Sarath Ajay Kumar, Anil Kumar Pidikiti, Vaidyanath Gottumukkula

**Affiliations:** 1 Department of Psychiatry, Siddhartha Medical College, Andhra Pradesh, India; 2 Department of Psychiatry, Government Medical College, Anantapur, India; 3 Department of Psychiatry, MNR Medical College & Hospital, MNR Nagar, Fasalwadi, Narsapur Road, Sangareddy-502294, India

**Keywords:** ICU psychiatry, psychiatric comorbidity, depression, anxiety, critical care, India

## Abstract

Psychiatric morbidity among ICU patients at a general hospital in India is of interest. Psychiatric diagnosis was assessed using the Mini International Neuropsychiatric Interview (MINI), while illness severity was measured with the APACHE-II scale for 400 ICU patients. The results revealed that, 65.5% prevalence of psychiatric disorders were 2 times higher than the primary care settings 23-42.5% and the parameters like mood 28.2% and anxiety 24.5%. Disorders predominated, with 72.4% prevalence in CNS patient's vs 42.1% in others and the results also showed that, there was 82.3% morbidity in >70yr vs 36.4% in <30yr (p<0.001). These findings mandate protocolized psychiatric screening for ICU patients, particularly targeting elderly males, those with CNS/respiratory disorders and APACHE-II scores >30. The research findings had suggested that, implementing brief MINI assessments during ICU rounds could significantly improve the detection and management of comorbid mental health conditions in critical care settings.

## Background:

Psychiatric disorders in intensive care unit (ICU) patients represent a significant yet under recognized challenge in modern healthcare. These conditions may emerge either as direct consequences of critical illness or as independent comorbidities, each carrying distinct implications for diagnosis and management. Globally, studies report that 20-60% of hospitalized patients experience psychiatric symptoms requiring specialist intervention, with particularly high rates observed in ICU settings [1, 2-3]. This psychiatric burden substantially impacts patient outcomes, contributing to prolonged hospital stays, increased healthcare costs and poorer long-term recovery [4]. The ICU environment itself serves as a potent trigger for psychological distress. Procedures like mechanical ventilation sleep disruption and physical restraints frequently precipitate acute stress reactions, with up to 60% of survivors developing post-traumatic stress symptoms [5-6]. Delirium, occurring in 80% of ventilated patients, represents an especially grave concern, associated with 2-3 times higher mortality risk and long-term cognitive impairment [7-8]. These psychiatric complications often persist beyond hospitalization, significantly diminishing patients' quality of life and functional recovery [3-4]. In the Indian context, several unique challenges exacerbate this problem. Our healthcare system contends with severe psychiatrist shortages (1:250,000 population), particularly in rural areas [9-10] (NMHS, 2016). Cultural factors [11] further complicate detection, as patients frequently express psychological distress through somatic complaints. Additionally, only 12% of intensivists receive formal psychiatric training leading to underdiagnosis of conditions that could otherwise be managed effectively [12]. Current research on ICU psychiatry reveals significant gaps in understanding the mental health challenges faced by critically ill patients in developing nations, as most existing studies predominantly focus on Western populations while neglecting crucial contextual factors [5, 13]. The literature largely fails to account for the profound influence of sociodemographic variables such as education levels, rural/urban disparities and cultural health beliefs that shape both the manifestation and recognition of psychiatric symptoms in low-resource settings [14]. Furthermore, there remains a striking paucity of research examining how specific medical conditions-particularly those prevalent in developing countries like infectious diseases or complications of uncontrolled chronic illnesses - may differentially impact psychiatric outcomes in ICU patients [12, 13]. These evidence-based insights will guide the creation of targeted training programs to enhance mental health detection capabilities among ICU staff [11- 12] focusing on recognizing psychiatric manifestations in critically ill patients while accounting for local cultural contexts. Therefore, it is of interest to evaluate psychiatric morbidity among ICU patients at a general hospital in India.

## Materials and Methods:

## Study design:

The present study is a cross-sectional observational study.

## Study place:

The study was conducted in the Intensive Care Units (ICUs) of Maharajah's Institutes of Medical Sciences general hospital in Nellimarla.

## Sample size:

We selected 400 patients for this study based on specific criteria.

## Study period:

The study was conducted for a period of one year and six months, from January 2018 through June 2019.

## Inclusion criteria:

[1] Informed consent: Patients were required to provide written consent to participate in the study.

[2] Demographics: Participants could be male or female and must have been at least 18 years old.

[3] ICU Stay: Patients had to have spent a minimum of 24 hours in the ICU.

## Exclusion criteria:

To ensure the accuracy of our data, we excluded patients without a reliable informant who could provide information on their behalf.

## Methodology:

In this study, we utilized several well-established instruments to ensure accurate data collection and analysis. Each tool was selected to fit the specific needs of our research objectives. Before commencing the study, we obtained ethical clearance from the Ethics Committee of the Medical Institute. All participants provided written informed consent, ensuring they understood the research's purpose and scope. A self-designed preform was used to gather basic demographic information about the patients. This included details such as name, age, sex, marital status and details about the informant, such as their name, relationship to the patient and the reliability of the information they provided. Modified BG Prasad Scale for Socio-Economic Status. The Modified BG Prasad Scale is an income-based tool used to assess socioeconomic status. It categorizes individuals into upper, upper-middle, middle, lower-middle and lower classes based on per capita monthly income: Upper Class: ≥6254; Upper-Middle Class: 3127-6253; Middle Class: 1876-3126; Lower Middle Class: 938-1875; Lower Class: <938. This scale is widely applicable, making it suitable for both urban and rural settings [15]. Mini International Neuropsychiatric Interview Schedule (MINI). The MINI is a widely validated, structured diagnostic interview developed to assess psychiatric disorders according to DSM-IV and ICD-10 criteria. It is lauded for its efficiency, with an average administration time of 18.7 minutes. In our study, the MINI was used to screen for psychiatric morbidity among ICU patients [16]. APACHE-II (Acute Physiology and Chronic Health Evaluation) Scale APACHE-II is a severity-of-disease classification system used to assess ICU patients' conditions within the first 24 hours of admission. It computes a score from 0 to 71, with higher scores indicating more severe disease and a greater risk of mortality. This scale was employed in our study to evaluate the severity of physical illness [17].

## Statistical analysis:

The data was analyzed using SPSS software version 25.0. Descriptive analyses were conducted on all variables to provide an overview of participant characteristics. For continuous data, mean and standard deviation were used to summarize and describe the central tendency and dispersion of the variables. Chi-square tests, including Pearson's and Fisher's exact tests, were utilized to compare categorical variables and assess any statistically significant associations. p-value less than 0.05 was adopted as the threshold for determining statistical significance in all analyses.

## Results:

A total of 400 ICU patients participated in this study. Among 400 patients studied, 66.5% (266) were male, with the majority (47.8%, 191) aged 51-70 years. Slightly over half (51.2%, 205) were from urban/semi-urban areas, while 55.8% (223) were illiterate. Most participants (87.5%, 350) were married and 73.3% (293) belonged to the middle socioeconomic class. Only 7.8% (31) had upper socioeconomic status and 19% (76) were from the lower class. The majority of participants experienced a major depressive episode, affecting 15.5% of the group (62 people), with 16.16% of males (43) and 14.18% of females (19) impacted. Following this, generalized anxiety disorder (GAD) was the next most common issue, affecting 14.5% of participants (58 people), including 15.8% of males (42) and 11.94% of females (16). Psychiatric morbidity was significantly higher in patients aged 51-70 years (p < 0.001) and among males (p = 0.001). Illiterate patients and those widowed/divorced also showed greater morbidity (p = 0.011 and p = 0.001, respectively). While urban/semi-urban residents had slightly higher rates, this difference lacked statistical significance (p = 0.187). Socioeconomic status did not significantly influence morbidity (p = 0.752), though middle-class patients were most affected. Figure 1 (see PDF) depicts the relationship between the physical disorders and psychiatric morbidity. Psychiatric morbidity was significantly linked to CNS, respiratory and gastrointestinal disorders (p < 0.001), but not to cardiovascular, renal, or other physical conditions (p > 0.05). Mood disorders were most common in CNS, cardiovascular and other physical illnesses, while anxiety disorders appeared more in respiratory and renal diseases. Substance use disorders (SUDs) were notably higher in patients with gastrointestinal problems. Figure 2 (see PDF) depicts the relationship between severity of physical illness and psychiatric morbidity. Patients with more severe physical illness (higher APACHE-II scores) showed significantly greater psychiatric morbidity (p = 0.004). Interestingly, anxiety disorders were more common in less severe cases (APACHE-II ≤19), while mood disorders predominated in sicker patients.

## Discussion:

This comprehensive study reveals critical insights about psychiatric morbidity in ICU patients, with several important findings emerging from the data. The overall prevalence of psychiatric disorders was strikingly high at 65.5%, significantly exceeding rates reported in general hospital or primary care settings (20-42.5%) [1, 9- 10]. This substantial difference likely reflects both the unique stressors of critical illness and our study's inclusive approach of assessing patients regardless of their physical health status [1] unlike some previous studies that only evaluated stabilized patients. The most common psychiatric manifestations were mood disorders (28.2%) and anxiety disorders (24.5%) [9, 10]. Consistent ith exists literature on medically ill populations, though our rates were notably higher than some comparable studies, particularly for mood disorders [3, 5 and 11]. Several demographic factors showed significant associations with psychiatric morbidity. Age demonstrated a particularly strong relationship, with prevalence increasing steadily to peak at 82.3% in patients over 70 years old [1, 2 and 17]. This linear age-risk relationship contrasts with some previous studies that found no association or even reduced risk in elderly populations 10, 11, possibly because our ICU setting revealed underlying vulnerabilities that might be masked in less acute medical contexts. Gender differences also emerged strikingly, with males showing higher overall psychiatric morbidity (71.05%) than females (54.47%) [1, 13], reversing typical gender patterns seen in community studies. This unexpected finding may reflect both the types of conditions leading to ICU admission in males and potential gender differences in coping with acute medical stress [7, 11]. Education level and marital status also proved significant factors. Illiterate patients had substantially higher psychiatric morbidity (70.85%) than literate patients (58.75%) [18, 19] likely reflects both the stress of navigating complex medical systems without education and potential differences in symptom reports. Marital status showed perhaps the most dramatic differences, with widowed or divorced patients experiencing the highest rates of psychiatric morbidity (82.3%) [20]. Followed by married patients (67.4%) and unmarried patients showing the lowest rates (36.4%). This gradient strongly suggests the protective role of social support during critical illness, while also hinting that the complete absence of marital ties might paradoxically confer some resilience, possibly through greater self-reliance. The study revealed important variations by medical diagnosis as well. Patients with CNS disorders showed the highest psychiatric morbidity (72.4%) [21]. Particularly mood disorders, likely reflecting both the psychological impact of neurological impairment and potential direct brain effects on emotional regulation. Respiratory system disorders were strongly associated with anxiety (65.8%) [22, 23]. Possibly due to distressing subjective experience of breathing difficulties. Gastrointestinal disorders showed a distinct pattern with higher rates of alcohol-related disorders [23], reflecting known epidemiological links between alcohol use and GI diseases. Illness severity, as measured by APACHE-II scores [6, 7 and 23], showed a clear relationship with psychiatric morbidity, increasing steadily until very high scores where mortality might obscure detection. This pattern suggests that psychological distress tracks with physical illness severity up to a point, after which extremely ill patients may become too physically compromised to manifest or report psychiatric symptoms. The peak psychiatric morbidity at APACHE-II scores of 30-34 (76.9%) provides a clinically useful marker for identifying patients at the highest risk. Several findings challenge conventional assumptions. The U-shaped relationship with socioeconomic status [15], where middle-class patients showed slightly higher morbidity than either lower- or upper-class patients, contradicts simple linear models of deprivation and mental health. Similarly, the lack of significant urban-rural differences despite trends toward higher rural morbidity [24] suggests that geographic factors may be less important than other social determinants in this context. These nuances highlight the complex interplay of biological, psychological and social factors in critical care settings. The study's findings have important clinical implications. The high overall prevalence argues for routine psychiatric assessment in ICU patients, particularly targeting high-risk groups like elderly patients, those with CNS or respiratory diagnoses and patients with limited social support. The system-specific patterns suggest tailored approaches might be needed - for instance, greater focus on mood symptoms in neurological patients versus anxiety management in respiratory cases. The strong association with illness severity underscores the need for integrated care that addresses both physical and mental health throughout the treatment course. While providing valuable insights, the study also raises important questions for future research. The unexpected gender and socioeconomic patterns warrant deeper investigation into cultural and systemic factors influencing mental health in critical care. The variations by medical diagnosis suggest value in exploring underlying mechanisms, potentially including inflammatory markers or specific neural pathways [22]. Longitudinal follow-up could help distinguish transient stress responses from persistent psychiatric conditions. The findings collectively argue for viewing ICU psychiatric morbidity as a multifaceted phenomenon requiring biopsychosocial approaches to both understanding and treatment [4, 12].

## Conclusion:

There is a need for an immediate change in ICU care like routine mental health screenings, tailored interventions for high-risk groups like elderly or neurologically compromised patients and closer liaison between intensivists and psychiatrists. By adopting such a bio-psycho-social approach, we can decrease the burden and effects of critical illness-shortening hospital stays, improving recovery and ultimately making ICUs not just life-saving but healing spaces for both body and mind.
